# Microfluidic Synthesis of Magnetic Silica Aerogels for Efficient Pesticide Removal from Water

**DOI:** 10.3390/gels11060463

**Published:** 2025-06-17

**Authors:** Dana-Ionela Tudorache (Trifa), Adelina-Gabriela Niculescu, Alexandra-Cătălina Bîrcă, Denisa Alexandra Florea, Marius Rădulescu, Bogdan-Ștefan Vasile, Roxana Trușcă, Dan-Eduard Mihaiescu, Tony Hadibarata, Alexandru-Mihai Grumezescu

**Affiliations:** 1 Department of Science and Engineering of Oxide Materials and Nanomaterials, National University of Science and Technology POLITEHNICA Bucharest, 1-7 Polizu Street, 011061 Bucharest, Romaniaalexandra.birca@upb.ro (A.-C.B.); denisa.florea@upb.ro (D.A.F.); roxana.trusca@upb.ro (R.T.); tony.hadibarata@upb.ro (T.H.); grumezescu@yahoo.com (A.-M.G.); 2 Research Institute of the University of Bucharest—ICUB, University of Bucharest, 90-92 Panduri, 050663 Bucharest, Romania; 3 Department of Inorganic Chemistry, Physical Chemistry and Electrochemistry, National University of Science and Technology POLITEHNICA Bucharest, 1-7 Polizu Street, 011061 Bucharest, Romania; marius.radulescu@upb.ro; 4 Research Center for Advanced Materials, Products and Processes, National University of Science and Technology POLITEHNICA Bucharest, 060042 Bucharest, Romania; bogdan.vasile@upb.ro; 5 National Research Center for Micro and Nanomaterials, National University of Science and Technology POLITEHNICA Bucharest, 060042 Bucharest, Romania; 6 Department of Organic Chemistry, National University of Science and Technology POLITEHNICA Bucharest, 1-7 Polizu Street, 011061 Bucharest, Romania; danedmih@gmail.com; 7 Environmental Engineering Program, Faculty of Engineering and Science, Curtin University Malaysia, CDT 250, Miri 98009, Malaysia

**Keywords:** microfluidic synthesis, silica-based aerogel, magnetic composite, pesticide removal, environmental applications

## Abstract

Aerogels have gained much interest in the last decades due to their specific properties, such as high porosity, high surface area, and low density, which have caused them to be used in multiple and varied fields. As the applicability of aerogels is tightly correlated to their morpho-structural features, special consideration must be allocated to the fabrication method. An emerging technique for producing nanostructured materials with tailored morphology and dimensions is represented by continuous-flow microfluidics. In this context, this work explores the synergic combination of aerogel-based materials with microfluidic synthesis platforms to generate advanced nanocomposite adsorbents for water decontamination. Specifically, this study presents the novel synthesis of a magnetic silica-based aerogel using a custom-designed 3D microfluidic platform, offering enhanced control over nanoparticle incorporation and gelation compared to conventional sol–gel techniques. The resulting gel was further dried via supercritical CO_2_ extraction to preserve its unique nanostructure. The multi-faceted physicochemical investigations (XRD, DLS, FT-IR, RAMAN, SEM, and TEM) confirmed the material’s uniform morphology, high porosity, and surface functionalization. The HR-MS FT-ICR analysis has also demonstrated the advanced material’s adsorption capacity for various pesticides, suggesting its adequacy for further environmental applications. An exceptional 93.7% extraction efficiency was registered for triazophos, underscoring the potential of microfluidic synthesis approaches in engineering advanced, eco-friendly adsorbent materials for water decontamination of relevant organic pollutants.

## 1. Introduction

In recent years, developments in nanotechnology have grown constantly, especially in manufacturing different nanoscale materials of interest for various advanced applications. The emergence of microfluidic devices solves some of the major limitations associated with classical nanomaterials synthetic methods. Microfluidic technology enables controllable manipulation and processing of small liquid volumes (i.e., ranging from 10^−9^ to 10^−18^ L), overcoming the limitations of chemical and physical conventional methods through their compact dimensions and precise operation. In the continuous-flow model, effective control over particle growth can be obtained in a simple manner, with little reagent usage and less environmental pollution than with traditional techniques [1,2,3,4,5,6,7,8].

Magnetic nanoparticles (MNPs) have gained significant attention among all synthesized and explored nanomaterials due to their versatility and attractive characteristics. When exposed to an external magnetic field, MNPs manifest direct interaction, making them unique materials that can be utilized in various applications [6,9,10,11]. Iron oxide nanoparticles (Fe_3_O_4_), one of the most investigated types of magnetic nanoparticles, have gained much interest in various domains because of their unique properties, biocompatibility, low toxicity, environmental safety, as well as cost-effectiveness [12,13,14]. Moreover, if the dimensions of the particles are small enough, ranging between 10 and 100 nm, they display superparamagnetism through their extremely short magnetic relaxation response time [15,16,17,18].

In addition to all the excellent characteristics of iron oxide nanoparticles, some challenges exist, such as sensitivity to oxidation, fast transformation to maghemite, and the tendency to agglomerate. The nanoparticles are usually coated or functionalized with inorganic and/or organic compounds to mitigate these adverse effects. In order to protect magnetite from aggregation and to increase its biocompatibility and stability, an organic modification is required. On the other hand, functionalization with inorganic compounds can enhance the catalytic activity and strengthen their stability or protect them from corrosion [6,19].

Environmental contamination is a significant issue resulting from various areas of industrialization and urbanization. The extensive and consistent utilization of pesticides in agriculture leads to a substantial rise in pesticide concentration levels. All of this contamination affects vegetables, fruits, soil, and water, and can threaten human health [20,21,22,23].

A potential technology that has recently gained much interest in being a proposal for various experimental techniques is solid-phase extraction (SPE). SPE is an effective sampling pretreatment and clean-up method for the successful extraction of numerous sample matrices [24,25,26,27]. It has undergone significant advancements and is now widely employed as a standard method in sample preparation procedures for both liquid and solid matrices. This approach is known for its simplicity, high recovery rates, minimal solvent usage, adaptability, potential for automation, and energy- and time-saving nature [26,28].

In recent years, many scientists have reported different and advanced materials, which have resulted in new and high-performance materials with applications in pollutant removal [29]. Various aerogel types, such as carbon, silica, metal oxide, metal–organic, and inorganic–organic hybrid aerogels, have been utilized in SPE due to their advantageous features, including large surface area, high porosity, and low density [30,31,32,33]. Silica-based aerogels are a unique class of mesoporous materials renowned for their exceptionally low density, high surface area, tunable porosity, and thermal and chemical stability. Typically synthesized via the sol–gel process, these materials undergo hydrolysis and condensation of silane precursors, followed by drying methods such as supercritical drying, ambient pressure drying, or freeze-drying to preserve their porous network [32,34,35,36].

The unique structural characteristics of silica aerogels have led to their utilization in diverse fields. Their thermal insulation properties are leveraged in the aerospace and construction industries [35,37,38]. Additionally, their integration into composites with carbon nanostructures has opened avenues in energy storage and electronic applications [38,39]. In environmental remediation, they serve as effective adsorbents for pollutants due to their high surface area and tunable surface chemistry [39]. The hydrophobic nature of silica and composite aerogels has allowed them to be used in several fields, permitting them to adsorb organic pollutants, such as POPs, or different inorganic contaminants, without compromising the porosity and network architecture [32,40].

There are various methods to obtain aerogel-based materials, such as sol–gel [41], self-assembly [42], epoxy crosslinking [43], emulsion coagulation [44], or 3D printing [45], each with its advantages, disadvantages, and material specificity. However, these methods are often time-consuming and high-cost, require post-processing, and may lead to impure aerogels [32]. Hence, renewed research interest has been directed towards improving aerogel fabrication and implementing new techniques to overcome existing challenges [36]. 

In this regard, our study proposes a novel aerogel synthesis route, implying the use of a custom-made microfluidic platform. By using a 3D vortex multilayered microreactor (priorly tested for Fe_3_O_4_-salicylic acid nanoparticle (Fe_3_O_4_-SA NP) production [9]), we obtained magnetite-incorporated silica-based aerogel nanocomposites in a well-controlled and rapid manner. Further, the developed nanostructured materials’ applicability was demonstrated for removing organic pollutants (i.e., pesticides) from aqueous samples, highlighting the great potential of these microfluidic-obtained aerogels for water decontamination.

## 2. Results and Discussion

### 2.1. Nanoparticle Characterization

Information about the hydrodynamic diameter and zeta potential of microfluidic-synthesized magnetite-based materials was obtained through DLS analysis. The results are summarized in Table 1. It can be observed that the value of the zeta potential is 61 mV, which is correlated with the dispersibility and colloidal stability of iron oxide, suggesting excellent electrostatic stabilization and minimal risk of aggregation in aqueous dispersion. Thus, the obtained materials are suitable for homogeneous incorporation into silica-based aerogels, where uniform dispersion in the sol–gel precursor is crucial for nanocomposite fabrication. The high positive potential indicates effective salicylic acid functionalization, offering steric and electrostatic stabilization. Moreover, the hydrodynamic diameter value (i.e., 92 nm) is also an excellent parameter that is correlated with small, functionalized magnetite NPs with no significant aggregation. The low standard deviation (i.e., 0.40 nm) suggests a narrow size distribution, the sample of analyzed particles being highly monodispersed.

**Figure 1:**
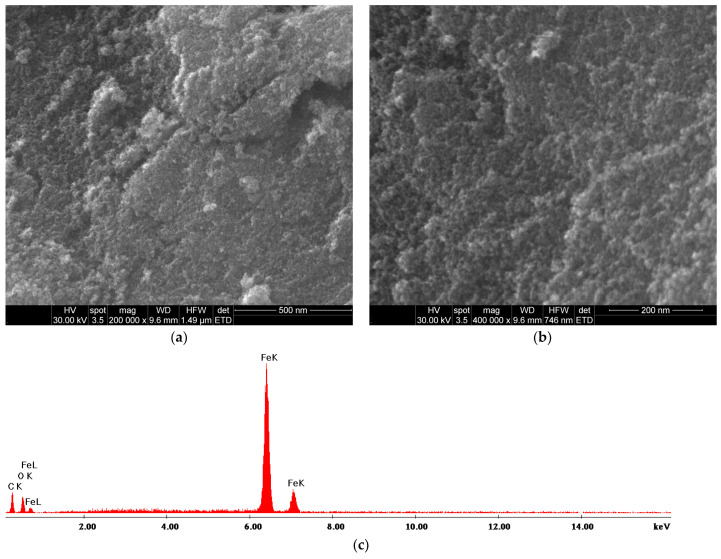
SEM micrographs at (**a**) 200,000× and (**b**) 400,000× magnification, and (**c**) EDS results for Fe_3_O_4_-SA NPs.

The structural and morphological characteristics of the Fe_3_O_4_-SA NPs were examined using SEM analysis. Figure 1 illustrates the uniform size distribution of synthesized nanoparticles and their quasi-spherical shape, which is common for magnetite nanoparticles. A slight tendency to agglomerate is also noticed in the micrographs, but without large-scale aggregates. The peaks for Fe and O were found in the EDS elemental composition analysis, suggesting successful magnetite formation.

**Figure 2:**
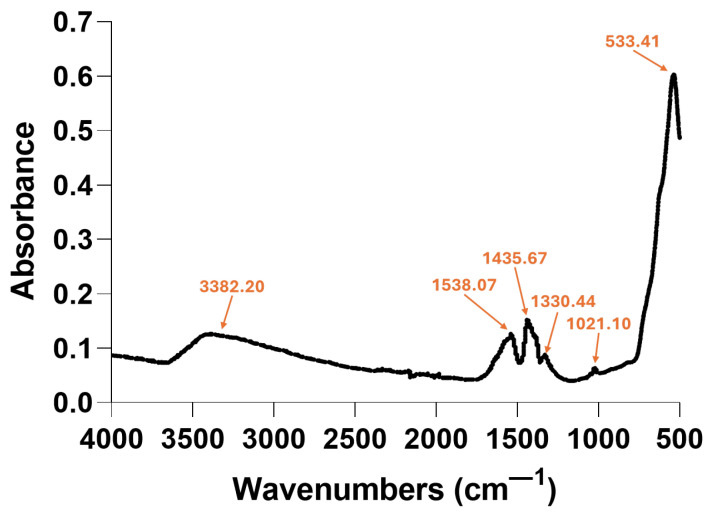
FT-IR spectra of Fe_3_O_4_-SA NPs.

FT-IR spectroscopy was performed to identify the functional groups present in the nanoparticle sample (Figure 2). The peak observed at ~533 cm^−1^ is the fingerprint for Fe–O stretching vibrations [46,47], confirming the presence of iron oxide nanoparticles. The broad peak identified at ~3382 cm^−1^ corresponds to the hydroxyl group stretching vibration, attributed to the -OH groups from the surface of Fe_3_O_4_ and/or salicylic acid (SA) coating [48]. Further, the peaks present at 1538, 1435, 1330, and 1021 cm^−1^ are consistent with the aromatic ring, carboxylic acid, and phenolic hydroxyl functionalities of SA [9], confirming successful surface functionalization.

The microfluidic platform has been previously reported in our recent studies [9,48,49], being demonstrated as an advantageous device for the production of uniform spherical Fe_3_O_4_-SA particles with nanometer dimensions and a narrow size distribution. The magnetic nanoparticles obtained in this study confirm the reproducibility of this microfluidic synthesis route, leading to similar results in terms of morpho-structural features for the Fe_3_O_4_-SA NPs.

### 2.2. Aerogel Characterization

Considering the advantages associated with microfluidic technology in general, and in particular with our three-dimensional multilayer vortex-mixing chip, we built upon the successful nanoparticle production and further explored the platform for the fabrication of advanced nanostructured composites. Thus, the platform was herein tested for synthesizing silica aerogel incorporated with iron oxide nanoparticles. The proposed method was proven effective, with the microfluidic flow control and enhanced mixing enabling the production of silica-based frameworks with uniformly dispersed functionalized magnetic nanoparticles within the gel matrix.

**Figure 3:**
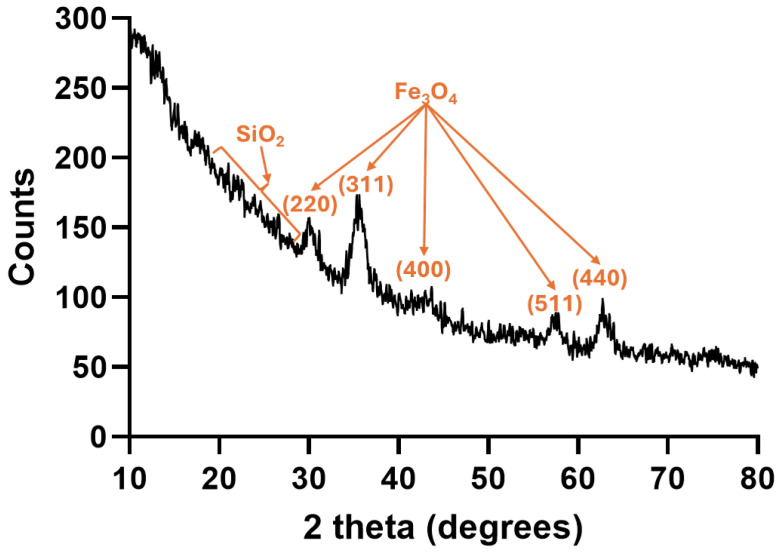
XRD diffractogram of magnetic silica-based aerogel composite.

The XRD analysis (Figure 3) of the obtained composite material shows the specific diffraction peaks for both iron oxide and silica. Thus, the diffractogram confirms the successful integration of crystalline Fe_3_O_4_ nanoparticles into an amorphous silica aerogel matrix, preserving both the magnetic properties (from the Fe_3_O_4_ crystalline phase) and the porous, disordered nature (from the silica phase). Specifically, the amorphous silica is visible through the broad hump around 20–30° while the sharp diffraction peaks overlaid on the broad background correspond to the crystalline structure of integrated Fe_3_O_4_-SA NPs. Moreover, the main peaks identified at 2theta angles of ~30.1°, ~35.5°, ~43.2°, ~57.1°, and ~62.7° correspond to the (220), (311), (400), (511), and (440) planes of magnetite with cubic inverse spinel structure (space group: Fd3̅m).

**Figure 4:**
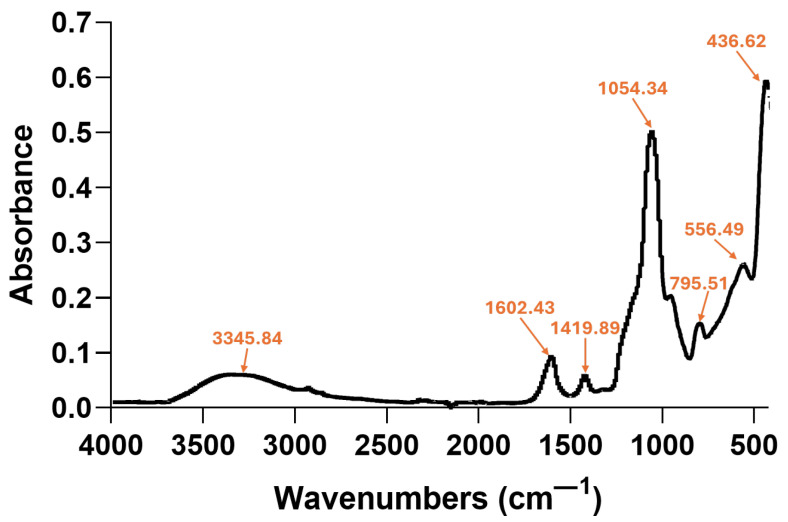
FT-IR spectrum of magnetic silica-based aerogel composite.

The FT-IR spectrum of the aerogel-based magnetic system obtained via a microfluidic device is shown in Figure 4. The peaks identified at ~3345, 1602, and 556 cm^−1^ are characteristic of -OH, COO^−^, and Fe-O functionalities, respectively, being found at slightly shifted wavenumbers compared to the spectrum of Fe_3_O_4_-SA NPs. These shifts reflect new hydrogen bonding and coordination environments, likely due to surface interactions with Si-OH groups and possible formation of Fe–O–Si bonds or interfacial rearrangement [48,50]. The additional new peaks, at ~1054 and ~795 cm^−1^, are attributed to Si–O–Si asymmetric and symmetric vibrations, respectively [51,52], validating the formation of the aerogel matrix.

**Figure 5:**
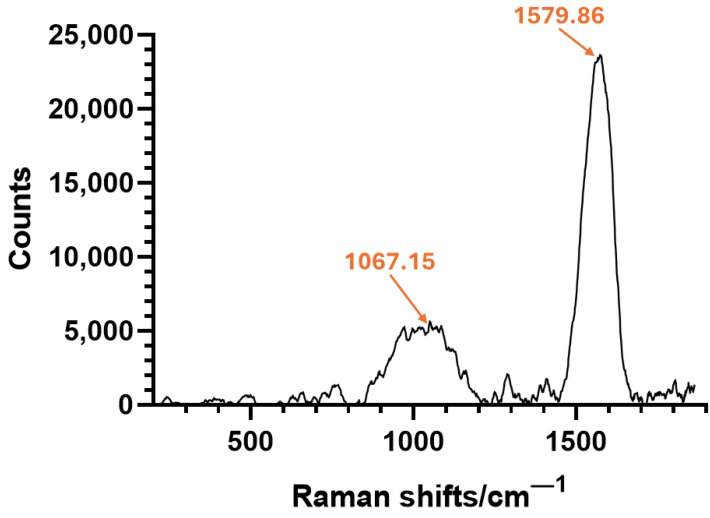
RAMAN spectrum of magnetic silica-based aerogel composite.

The RAMAN spectroscopy analysis, illustrated in Figure 5, was conducted to complementarily investigate the key functional groups present in the composite silica aerogel. The most prominent peak observed at ~1580 cm^−1^ corresponds to the organic compound (i.e., salicylic acid) used for iron oxide nanoparticle functionalization, being attributed to the aromatic ring stretching mode (C=C) [53]. This confirms that SA functionalization maintains chemical integrity after successfully integrating Fe_3_O_4_-SA NPs into the silica matrix. On the other hand, the broad peak near 1100 cm^−1^ belongs to Si–O–Si stretching (ref. [54]) of the silica aerogel framework, demonstrating the formation of the silica network. Additional minor features were observed around 765, 1287, and 1387 cm^−1^, consistent with organosilicon and aromatic functional groups.

DLS results obtained for the magnetic silica-based aerogel composite are summarized in Table 2. The investigation revealed a substantial increase in hydrodynamic radius (~4038 nm) compared to the parent Fe_3_O_4_-SA nanoparticles, confirming the formation of micron-sized clusters or fragments of the porous aerogel network. On the other hand, the zeta potential has a moderate negative value (i.e., –25.86 mV), suggesting partial colloidal stability and indicating that surface chemistry has changed (e.g., due to interaction with alginate or silica network). Although a high polydispersity and standard deviation indicate some degree of aggregation and structural heterogeneity, these values are typical for aerogel materials post-supercritical drying. This heterogeneity arises due to variations in pore size distribution and surface energy, which are influenced by the drying conditions [55].

**Figure 6:**
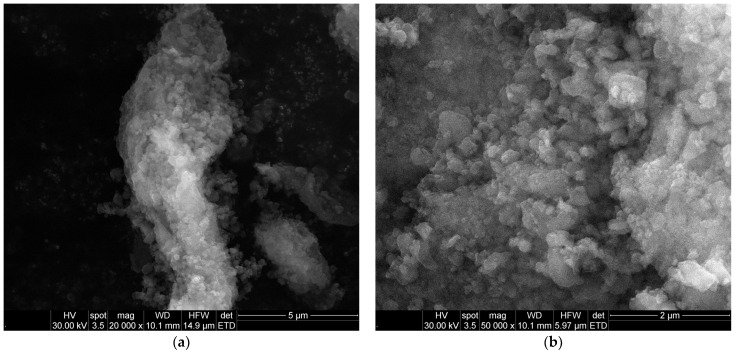

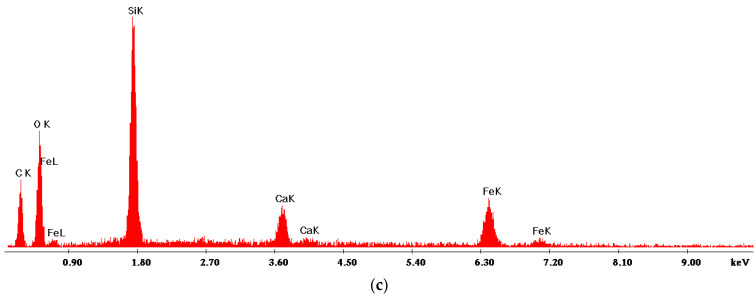
SEM micrographs at (**a**) 20,000× and (**b**) 50,000× magnification, and (**c**) EDS results for magnetic silica-based aerogel composite.

The SEM images (Figure 6a,b) reveal the typical porous microarchitecture of the aerogel, formed by a network of aggregated spherical and submicron domains. The open, sponge-like morphology and the presence of interconnected voids are characteristic of silica aerogels and contribute to their large surface area and diffusion accessibility. Some localized densification or clustering is observed, which is expected due to the incorporation of magnetic nanoparticles and the gelation dynamics. These features support the structural integrity and accessibility of the porous matrix for adsorption applications. In addition, the EDS elemental composition analysis (Figure 6c) confirms the presence of iron, oxygen, and silicon in the structure, which are correlated to the integration of iron oxide nanoparticles in the silica matrix. These findings support the structural and compositional integrity of the synthesized aerogel composite.

**Figure 7:**
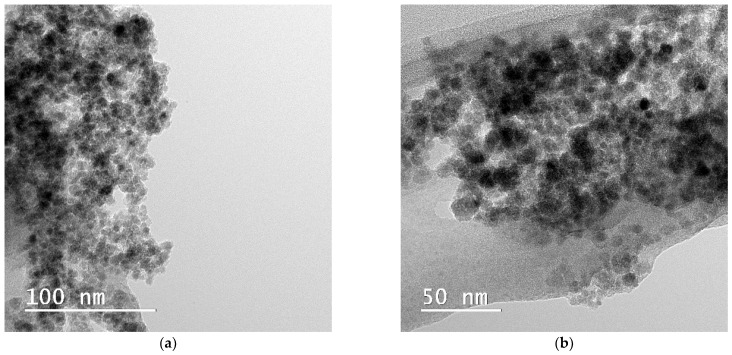

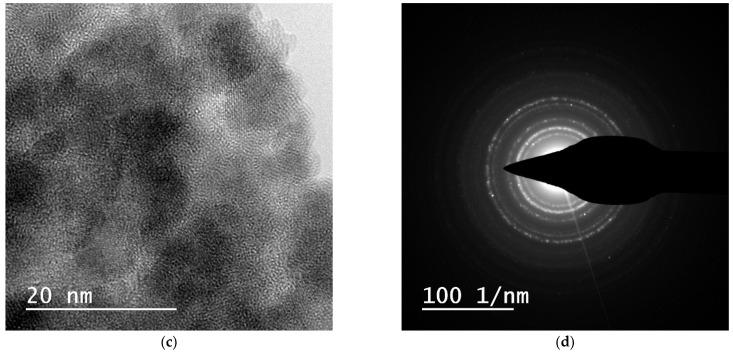
(**a,b**) Bright-field TEM images, (**c**) high-resolution TEM image, and (**d**) SAED pattern of magnetic silica-based aerogel composite.

The TEM images illustrated in Figure 7a,b show the granular microstructure and uniform distribution of the Fe_3_O_4_-SA nanoparticles, in agreement with SEM micrographs. The dark regions correspond to electron-dense magnetic nanoparticles, while the lighter background is typical of amorphous silica, which has low electron contrast. Thus, TEM analysis, including high-resolution TEM (Figure 7c), revealed the granular microstructure and confirmed the presence of Fe_3_O_4_-SA nanoparticles with sizes between 5 and 20 nm, uniformly distributed within the amorphous silica matrix. Even though the particles are well-dispersed, TEM micrographs reveal a slight tendency toward agglomeration. Further, the SAED pattern (Figure 7d) exhibits discrete concentric diffraction rings that correspond to specific lattice planes of inverse spinel Fe_3_O_4_. Specifically, the rings correspond to the (220), (311), (400), (511), and (440) planes of magnetite, confirming that the crystalline nature of Fe_3_O_4_ was retained throughout the synthesis and supercritical drying steps. These findings correlate with the XRD analysis, providing consistent crystallographic evidence across techniques.

As demonstrated by the FT-IR, XRD, TEM/SAED, and EDS analyses, Fe_3_O_4_-SA NPs retain crystallinity and are successfully embedded in the amorphous silica matrix. Moreover, scanning and transmission electron micrographs revealed an open network structure, a favorable morphology for adsorption through high surface area, accessibility of active sites, and facile diffusion of pollutant compounds. Additionally, the magnetic functionality and structural integration offer promising environmental remediation potential, improving the aerogel-based composite’s performance and recyclability.

### 2.3. Decontamination Capacity

The quantitative analysis of pesticide removal from contaminated water using the magnetic silica-based aerogel composite is summarized in Table 3. The data were obtained through high-resolution mass spectrometry (HR-MS FT-ICR) and reflect the material’s strong adsorptive capacity. The laboratory decontamination tests on various pesticides showed good adsorption capacity for the magnetic silica aerogel, which makes it an ideal candidate for further application in environmental remediation. The best results from the evaluated pesticides were registered for triazophos. This chemical compound, often present in insecticides, was removed with a ~93.7% extraction efficiency from the contaminated water sample.

The adsorption behavior of various pesticides on the magnetic silica aerogel can be rationalized through a combination of structural, electronic, and interfacial considerations. The aerogel features a highly porous silica network decorated with magnetite nanoparticles, which are surface-functionalized with salicylic acid. This modification introduces aromatic rings and hydroxyl/carboxyl groups, enhancing the aerogel’s affinity for polar and aromatic pollutants through several interaction modes.

Polarity and functional groups play a crucial role in dictating adsorption efficiency. For instance, triazophos, the best-performing pesticide (93.7% removal), contains multiple polar moieties (N=, P=S, P-O) and is relatively hydrophilic, promoting strong hydrogen bonding and electrostatic interactions with surface silanol groups and salicylic acid residues. In contrast, pesticides like trifluralin and chlorpropham, which have lower polarity and fewer electronegative atoms available for interaction, show reduced removal efficiencies.

The electronegativity of constituent atoms (particularly O, N, P, and S) enhances interactions via dipole–dipole attraction and potential coordination with Fe^3+^ centers on the magnetite surface. Pesticides with aromatic rings (e.g., fenson, tolclofos-methyl) may further engage in π–π stacking with the salicylate-modified magnetite, contributing to higher adsorption as observed in fenson (78.9%).

Moreover, the magnetite–salicylic acid interface acts as a multifunctional adsorptive domain. The hydrophilic carboxylate and hydroxyl groups of salicylic acid can form hydrogen bonds with polar pesticides, while the aromatic ring can stabilize adsorbates via non-covalent interactions. This hybrid binding landscape allows for both physical entrapment and specific chemical affinity, explaining the broad-spectrum yet selective adsorption performance.

For each pesticide, extraction efficiency was calculated using the following formula:

$$Extraction\;Efficiency\;\left(\%\right)=\frac{Initial\;conc.-Residual\;conc.}{Initial\;conc.}\times 100$$

### 2.4. Discussion

To the best of our knowledge, this is the first report of aerogel synthesis on a microfluidic platform. The custom-made continuous-flow 3D microfluidic platform with vortex passive mixing enabled us to fabricate a silica-based magnetic aerogel with precise control over nanocomposite composition and structure.

Concerning the raw materials used for obtaining aerogels, traditional silica precursors are tetraethyl orthosilicate (TEOS), tetramethyl orthosilicate (TMOS), their derivatives, and silicate salts [56]; however, TEOS and TMOS are the main silica precursors [35]. TEOS is less harmful and mild [57], while TMOS can attenuate the fracturing of the aerogel network during drying processes [58]. Our choice of sodium trisilicate was made considering the following advantages: cost-effectiveness, wide availability [59,60], and improved properties for the final product, such as low density, high porosity, and high specific surface area [61].

For the control of gelation, alginate was used as a gelation agent. It is a natural polysaccharide, non-toxic, environmentally friendly, and low-cost material, which makes it an excellent candidate for various applications [62], gaining interest in aerogel fabrication as well. For instance, Li et al. [63] utilized sodium alginate as a raw material to obtain an alginic acid carbon aerogel with the application as an adsorbent for oily dyes and contaminated aquatic systems, and the results show improvement compared to ferric alginate carbon and calcium alginate aerogels. In another study, El-Desouky et al. [64] combined a composite metal–organic framework and sodium alginate to improve adsorption capacity compared to using these two materials separately. The aerogel was used to remove food coloring from wastewater (e.g., Tartrazine), demonstrating excellent adsorption capacity.

Another important factor crucial for the aerogel’s final properties is the surfactant, which acts as a “template” for the structural framework [65]. Song et al. [66] obtained poly(methylmesosiloxane) aerogel short fibers using methyltrimethoxysilane and CTAB. The role of the surfactant was to protect the fiber structure through electrostatic repulsion, and the internal pore structure of the aerogel was precisely controlled by the aggregation state of CTAB micelles in water. Besides the surfactant’s role in inducing porosity, Xing et al. [67] also utilized CTAB to promote rapid gelation of feather keratin and silk fibroin porous aerogel.

Therefore, all employed materials to create the magnetic silica-based aerogel via the microfluidic platform were chosen due to their exceptional qualities and suitability for the envisaged environmental remediation application.

The major water contamination problem is attributable to human activities, such as waste resulting from hospitals, homes, chemical industries, agricultural production, and various other industrial fields, which negatively impact the environment and human health [68,69,70]. There are already multiple studies that have examined various aerogel types (e.g., carbon-based [71,72,73,74], cellulose-based [75], and polymer-based [76]) as adsorbents for removing organic contaminants, including pesticides, herbicides, or insecticides.

Regarding the silica-based aerogel utilized in environmental applications, some studies have been conducted on the extraction of pesticides [77,78], dyes [79,80,81,82], pharmaceuticals [83,84,85], and heavy metals [86,87]. All these research papers have demonstrated that silica aerogels, due to their unique properties such as porosity [88], high surface area [89], and the possibility of functionalization with organic or inorganic moieties during the synthesis [32], make them an excellent candidate for environmental purification. All these properties and advantages lead us to explore the adsorption capacity of silica aerogel on different pesticide compounds and to examine if the specific characteristics of silica aerogel synthesized via a microfluidic device are the same or enhanced compared with the traditional synthesis routes.

Therefore, our study focused on obtaining silica aerogel via the microfluidic technique and evaluating its adsorption capacity for extracting possible pesticides that might be present in the environment, especially in the water. The microfluidically synthesized composite showed high removal efficiencies across various pesticide classes, including organophosphates, carbamates, and dinitroanilines. Notably, triazophos—a widely used organophosphorus insecticide—was removed with an efficiency of 93.67%, confirming the high affinity of the composite for hazardous organic micropollutants. Hayat et al. [90] also investigated pesticide removal, using orange juice samples instead of contaminated water. The researchers developed ormosil, an amine-functionalized, organically-modified silica, which exhibited a good adsorption capacity of triazophos, with a 98.2% extraction efficiency. Thus, our aerogel composite’s triazophos extraction efficiency correlates with the abovementioned study, making the newly developed material an excellent candidate for water decontamination.

Regarding all the pesticide examples that are presented in Table 3, in the literature, only a few studies were performed on fenthion extraction using different graphene aerogels [91,92]. To our knowledge, the remaining pesticides presented in this study have not been previously extracted from contaminated water using aerogel-based materials. Besides triazophos, the proposed microfluidically synthesized magnetic aerogel nanocomposite has high performance against structurally diverse pesticides, displaying significant efficiencies for chlorthal-dimethyl (~63%), fenson (~79%), fenthion (~60%), mevinphos (~57%), and tolclofos-methyl (~57%).

These findings underscore the aerogel’s potential for real-world water purification and decontamination applications, particularly in the context of agricultural runoff and industrial pollution. The magnetic silica-based aerogel composite retained its monolithic structure and porosity during aqueous exposure, showing no signs of disintegration or collapse. Its robust architecture enabled efficient magnetic retrieval after the adsorption process, confirming the silica network’s stability and the functionality of the embedded Fe_3_O_4_ nanoparticles. The combination of structural integrity, adsorptive performance, and magnetic responsiveness positions this material as a promising candidate for reusable water treatment systems. Additionally, its fabrication from biocompatible precursors enhances its appeal for eco-friendly remediation. While the current work focused on single-use performance, future studies will explore recyclability and regeneration efficiency across multiple adsorption-desorption cycles to further assess its practical viability.

## 3. Conclusions

In this study, we report for the first time the synthesis of a magnetic silica-based aerogel composite via a custom-designed 3D microfluidic platform. This novel route enabled precise control over the dispersion and integration of salicylic acid-coated Fe_3_O_4_ nanoparticles into a porous silica matrix, using a continuous sol–gel process followed by supercritical CO_2_ drying. The resulting hybrid aerogel exhibited a highly porous, sponge-like architecture with well-preserved nanostructural features, as confirmed by SEM, TEM, and SAED analyses. The retention of the crystalline structure of Fe_3_O_4_ within the amorphous silica network, combined with high zeta potential and good dispersibility, confirms the physicochemical stability of the composite.

The material’s environmental applicability was demonstrated through HR-MS FT-ICR analysis, and the results show excellent removal efficiency for a range of pesticide contaminants. Triazophos obtained an outstanding removal capacity of 93.67% efficiency, positioning this nanostructured aerogel as a strong candidate for advanced water decontamination technologies. Integrating magnetic properties further enables potential magnetic-assisted separation and recyclability in future process designs.

Understanding the impact of pesticides on the environment and the adsorption capacity of the synthesized material, further investigations are necessary to fully evaluate the material’s long-term performance. Limitations such as potential nanoparticle leaching, regeneration capacity over multiple use cycles, and adsorption kinetics in dynamic flow systems remain to be addressed. Additionally, real wastewater matrices with complex pollutant loads must be tested to validate the composite’s efficiency under practical environmental conditions.

Looking forward, the microfluidic approach introduced here offers a scalable and tunable platform for engineering functional aerogels with tailored properties. This work opens new avenues for developing multifunctional materials that combine porosity, magnetic recovery, and chemical specificity, offering sustainable solutions for environmental remediation, particularly in the context of agricultural and industrial pollution.

## 4. Materials and Methods

### 4.1. Materials

Both synthesis processes (i.e., for obtaining Fe_3_O_4_-SA NPs and aerogel-based magnetic nanocomposites) were carried out within a three-dimensional multilayered polymethylmethacrylate (PMMA) microfluidic platform. The 3D vortex-mixing microreactor design and fabrication are detailed in our previous studies [9,48].

For the on-chip synthesis of iron oxide nanoparticles were utilized iron(III) chloride (FeCl_3_) and iron(II) sulfate heptahydrate (FeSO_4_ × 7H_2_O) acquired from Sigma Aldrich (Darmstadt, Germany) for the iron precursors solution, and sodium hydroxide NaOH (Lach-Ner, Tovarni, Czech Republic) dissolved in ultrapure water as precipitation environment. Salicylic acid (C_7_H_6_O_3_), purchased from Atochim Prod (Bucharest, Romania), was utilized to modify the surface of iron oxide nanoparticles.

To obtain the silica-based aerogel were used sodium trisilicate (Na_2_O_7_Si_3_), cetyltrimethylammonium bromide (CTAB^_^C_19_H_42_BrN), alginic acid sodium salt from brown algae, calcium chloride, and ammonium chloride (NH_4_Cl) purchased from Sigma Aldrich Merck (Darmstadt, Germany), acetic acid (C_2_H_4_O_2_) bought from Emsure Merck Millipore (Darmstadt, Germany), NaOH acquired from Lach-Ner, and the dispersed nanoparticles priorly obtained on-chip.

All the chemicals involved in the synthesis processes were of analytical grade and used as received. Ultrapure water was utilized throughout all experimental steps.

### 4.2. Methods

#### 4.2.1. Nanoparticle Synthesis

**Figure 8:**
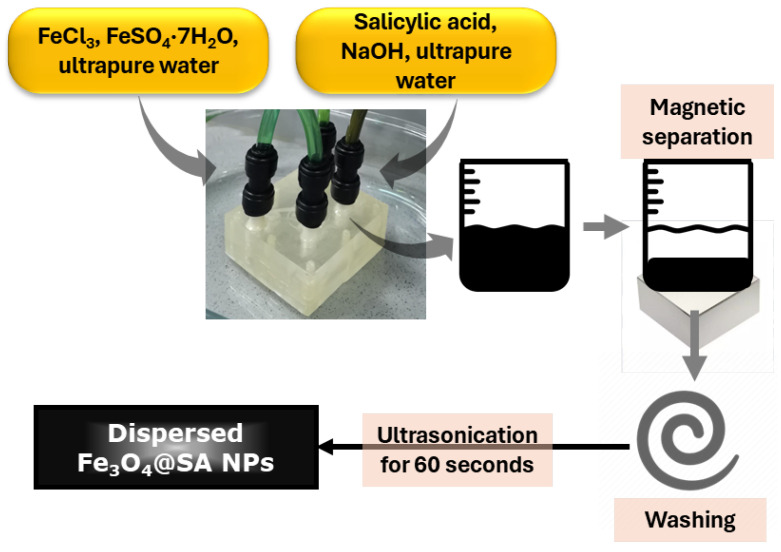
Schematic representation of Fe_3_O_4_-SA NP synthesis.

Fe_3_O_4_-SA NPs were prepared through on-chip co-precipitation, as depicted in Figure 8. Specifically, Fe^3+^ and Fe^2+^ ions dissolved in a 2 to 1 molar ratio in ultrapure water, and 2% sodium hydroxide and 1% salicylic acid dissolved in an equal amount of ultrapure water, were simultaneously introduced into the 3D vortex micromixer, as described in our previous study [49]. An osmotic pump (PSP 220 Pump, Model No. CAR6003, Water Quality Association, Lisle, IL, USA) was operated in order to inject the two solutions into the microfluidic platform. Within the microchannels, salicylic acid-functionalized Fe_3_O_4_ nanoparticles were formed as a black precipitate. The product was collected from the outlets of the microfluidic device, magnetically separated, and washed 5 times with ultrapure water in order to eliminate all the traces of unreacted reagents.

#### 4.2.2. Aerogel Synthesis

The silica precursor solution was made by dissolving 60 g of sodium trisilicate and 3.5 g of NaOH in 1000 mL of ultrapure water. The second solution contains 0.5 g of CTAB surfactant, 1 g of alginic acid, and 50 mL of dispersed magnetite (c = 0.45%) in a 300 mL total volume. Solution A was prepared by adding 150 mL of solution 1 and 150 mL of solution 2.

**Figure 9:**
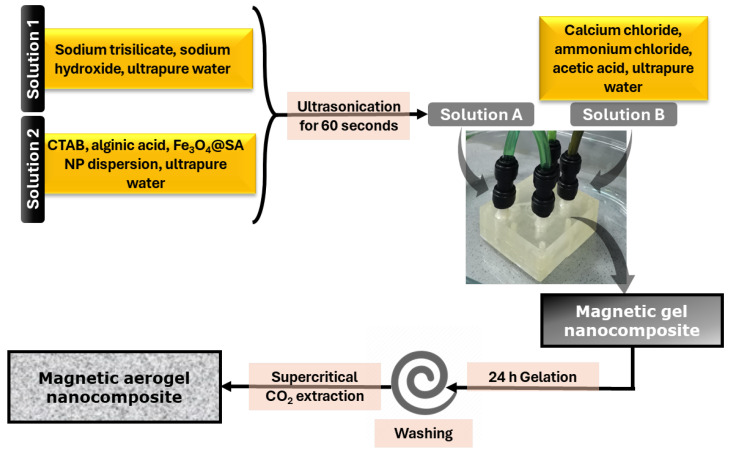
Schematic representation of magnetic aerogel nanocomposite synthesis.

Solution B consists of 20 g CaCl_2_ and 2 g NH_4_Cl dissolved in ultrapure water and 10 mL acetic acid, with a 400 mL total volume. First, 100 mL of ultrapure water and 100 mL of solution B were pumped into the osmotic pump, and then solutions A and B were pumped simultaneously. The obtained product was collected from the microfluidic platform outlets and allowed to stand at room temperature for 24 h in order to allow the gelation process to finish. The next day, the product was transferred into centrifuge tubes and washed several times with ultrapure water and ethanol. The next step involved supercritical CO_2_ extraction as the drying technique, which eliminated the solvent from the microporous structure of the silica aerogel. The overall process of on-chip preparation of the composite aerogel and processing steps are visually represented in Figure 9.

#### 4.2.3. Nanoparticle Characterization

Dynamic light scattering (DLS) analyses were performed using a DelsaMax Pro-type device equipped with a 532 nm laser. The powder samples were dispersed in ultrapure water and maintained at room temperature. All samples were subjected to ultrasound for 10 min to ensure optimal dispersion using an ultrasonic bath.

Morphological information regarding Fe_3_O_4_-SA NPs and aerogel-based magnetic nanocomposites was revealed using scanning electron microscopy (SEM) analysis. The powder samples were fixed on a carbon-bearing slide and placed in the analysis chamber of an Inspect F50 scanning electron microscope (Thermo Fisher–FEI, Eindhoven, The Netherlands). Micrographs were captured via a secondary electron beam scattering with a 30 keV energy and a spot size of 3.5. The elemental composition data were obtained with the help of the Energy Dispersive Spectroscopy (EDS) module, which was incorporated into the SEM system.

FT-IR analysis was further utilized to determine the presence of functional groups and confirm the composition of the samples. For this purpose, a Thermo iN10-MX FTIR spectrometer (Waltham, MA, USA) was used, with the collection being performed within the 4000–400 cm^−1^ range.

#### 4.2.4. Aerogel Characterization

X-ray diffraction (XRD) was performed to gather information on the phase composition and crystallinity of the aerogel-based magnetic nanocomposite. For the investigation, a PANalytical Empyrean model diffractometer (PANalytical, Almelo, The Netherlands), equipped with a 2xGe 220 hybrid monochromator on the incident side and a parallel plate collimator attached to PIXcel 3D detector on the diffracted side was used. Grazing incidence X-ray diffraction measurements were performed at room temperature, with an angle of incidence ω = 0.5° for Bragg angle values of 2θ between 10° and 80°, using Cu Kα radiation with λ = 1.5406 Å (40 mA and 45 kV).

A Thermo Fisher Scientific 80–200 Titan Themis transmission electron microscope (Hillsboro, OR, USA) was used to obtain high-resolution TEM micrographs. The microscope performs at 200 kV in transmission mode, with line and point of 2Å and 1Å, respectively. In addition, crystallographic data was obtained via the SAED module, which is integrated into the TEM system.

RAMAN spectroscopy (Renishaw inVia Raman microscope, Wotton-under-Edge, UK) was performed to explore material distribution within silica-based aerogel nanostructured composites. The measurements were conducted with a spatial resolution ranging from 0.25 to 4 μm. Spectra were collected using a 532 nm laser wavelength at a 50% laser intensity. For each sample, 40 scans were performed across a 200–1900 cm^−1^ Raman shift range.

SEM, EDS, FT-IR, and DLS analyses were also utilized to characterize the synthesized material from morphological and structural points of view, similar to those mentioned for the characterization of Fe_3_O_4_-SA NPs.

#### 4.2.5. Water Decontamination Study

**Figure 10:**
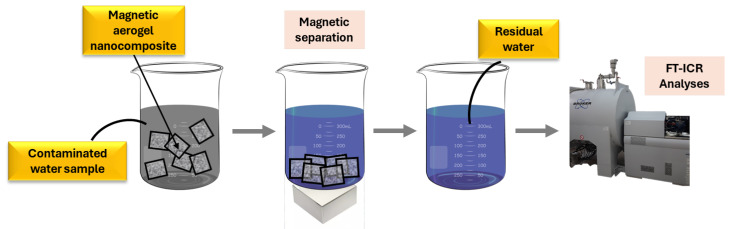
Schematic representation of water decontamination performance evaluation.

The extraction capacity of the magnetic aerogel composite was tested on ultrapure water samples spiked with pesticides at ppb levels. Aerogel powder was added to the pesticide-contaminated water sample, and the organic pollutants were allowed to be absorbed for 30 min (Figure 10). Water samples were evaluated before and after aerogel adsorption, registering the initial and residual concentrations of pollutants. This was possible through high-resolution mass spectrometry (HR-MS) analysis performed using a Fourier transform ion cyclotron resonance (FT-ICR) spectrometer equipped with a 15 T superconducting magnet (SolariX-XR, Bruker Daltonics, Bremen, Germany). The samples were introduced via direct infusion utilizing negative ionization (ESI) at a flow rate of 310 µL/h. The nebulization gas pressure (N_2_) was maintained at 1.5 L/min, while the dry gas flow rate was set to 2 L/min and a temperature of 210 °C. The spectra were collected over a mass range of 92 to 1500 amu and a source voltage of 4300 V.

## Figures and Tables

**Table 1:** DLS analysis of Fe_3_O_4_-SA NPs.

Zeta Potential (mV)	St. Dev. (mV)	Diameter (nm)	St. Dev. (nm)
61.271	1.26	92.288	0.40

Results are an average of three measurements.

**Table 2:** DLS analysis of magnetic silica-based aerogel composite.

Zeta (mV)	St. Dev.	Hydrodynamic Radius (nm)	St. Dev.
−25.86	1.73	4038.17	372.42

Results are an average of three measurements.

**Table 3:** Quantitative decontamination performance of the magnetic silica-based aerogel composite for various pesticide pollutants, assessed by HR-MS FT-ICR.

Pesticide	Molecular Formula	Chemical Structure	*m*/*z*	z	Resolution	Initial Conc. (ppb)	Residual Conc. (ppb)	Extraction Efficiency (%)
Chlorthal-dimethyl	C_10_H_6_Cl_4_O_4_	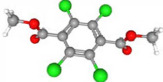	332.9061	1+	375,885	1.011	0.370	63.39
Chlorpropham	C_10_H_12_ClNO_2_	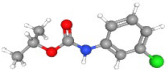	214.06289	1+	539,082	0.9925	0.507	48.93
Fenitrothion	C_9_H_12_NO_5_PS	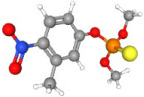	278.0245	1+	474,842	1.0095	0.629	37.68
Fenson	C_12_H_9_ClO_3_S	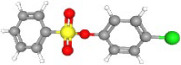	269.00327	1+	464,620	0.9976	0.210	78.94
Fenthion	C_10_H_15_O_3_PS_2_	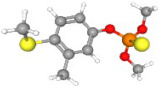	279.02709	1+	438,185	1.2664	0.505	60.12
Mevinphos	C_7_H_13_O_6_P	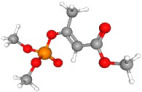	225.05167	1+	586,354	2.0058	0.860	57.12
Propyzamide	C_12_H_11_Cl_2_NO	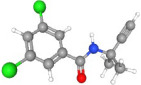	256.02891	1+	528,620	0.9931	0.647	34.84
Prothiofos	C_11_H_15_Cl_2_O_2_PS_2_	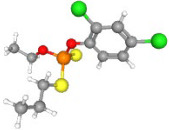	344.96992	1+	355,745	1.0059	0.518	48.48
Tolclofos-methyl	C_9_H_11_Cl_2_O_3_PS	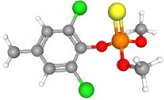	300.96150	1+	429,356	0.9907	0.424	57.21
Triazophos	C_12_H_16_N_3_O_3_PS	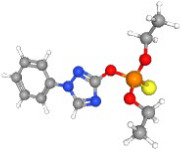	314.07211	1+	436,730	0.9948	0.063	93.67
Trifluralin	C_13_H_16_F_3_N_3_O_4_	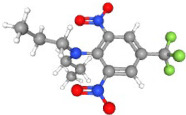	336.11638	1+	342,422	0.9893	0.538	45.62

Atoms: Grey–C, White–H, Neon Green–Cl, Red–O, Blue–N, Orange–P, Yellow–S, Lime Green–F.

## Data Availability

The original contributions presented in this study are included in the article. Further inquiries can be directed to the corresponding author.
